# Perceptions and Impact of Mandatory eLearning for Foundation Trainee Doctors: A Qualitative Evaluation

**DOI:** 10.1371/journal.pone.0168558

**Published:** 2016-12-22

**Authors:** Hannah L. Brooks, Sarah K. Pontefract, Hannah K. Vallance, Christine A. Hirsch, Elizabeth Hughes, Robin E. Ferner, John F. Marriott, Jamie J. Coleman

**Affiliations:** 1 College of Medical and Dental Sciences, University of Birmingham, Birmingham, United Kingdom; 2 University Hospitals Birmingham NHS Foundation Trust, Edgbaston, Birmingham, United Kingdom; 3 Health Education England, Edgbaston, Birmingham, United Kingdom; 4 West Midlands Centre for Adverse Drug Reactions, Sandwell and West Birmingham NHS Trust, Birmingham, United Kingdom; Waseda University, JAPAN

## Abstract

**Background:**

Junior doctors in the UK must complete various educational components during their two year Foundation training programme. It is important that mandatory learning is informative and engaging. The aim of this study was to evaluate trainee doctors’ perceptions of a Technology Enhanced Learning (TEL) programme developed to improve prescribing competency.

**Method:**

Focus groups and interviews were conducted at three hospital sites in the West Midlands. Codes, sub-themes and themes were determined using deductive and inductive thematic analysis.

**Results:**

Data were collected from 38 Foundation trainee doctors. Results revealed major themes relating to prescribing education, the user experience and user engagement. Key findings included the positive impact of preparedness following undergraduate education on the user experience of the TEL programme at the postgraduate level; the impact of content, structure, and individual learning needs and styles on the user experience; and the impact of motivation and time on engagement. Most trainees engaged with the programme owing to its mandatory nature; however, some trainees also used the programme voluntarily, for example, to acquire knowledge prior to starting a new placement.

**Conclusions:**

It is important to ensure that learners are willing to engage with mandatory TEL, and that they have the time and motivation to do so. It is also important to ensure that learners have a positive user experience and that in designing TEL individual differences in learning styles and needs are taken into account. These findings have implications for educators and system developers in the construction and design of mandatory eLearning programmes.

## Introduction

The Foundation training programme for UK junior doctors (those in their first two years after qualifying with a medical degree from university, equivalent to junior residency years; F1 and F2) involves a demanding schedule, including various mandatory educational components [1]. Foundation trainees are currently allocated three hours protected learning time during each working week in which they are encouraged to attend formal teaching sessions and/or complete mandated learning. An increasing amount of medical education is being delivered online [2, 3], utilising resources such as Technology Enhanced Learning (TEL). Various forms of TEL have been incorporated into medical education, including smartphone applications [4], flipped classrooms [5], and eLearning [6]. Such learning may be as effective as traditional face-to-face teaching methods and offers many benefits for the learner [7]. However, Foundation trainees do not always dedicate the appropriate amount of time to complete TEL [8], which may question learners’ preference for this approach [9]. As such, it is important to evaluate users’ satisfaction with and perceptions of TEL, in order to understand how to optimise engagement and ensure learning outcomes are achieved in the short amount of time allocated to such training.

There are many factors that can influence user engagement and achievement of learning outcomes via online learning platforms. For example, the Technology Acceptance Model (TAM; TAM-2; TAM-3) [10–12] posits that users’ attitudes towards the technology and subsequently their behavioural intention to use it determines their actual level of use. Ease of use and perceived usefulness of the technology impact upon users’ attitudes, while experience with the technology and voluntariness of use determine users’ behavioural intentions. Various other factors, such as subjective norms, image and job relevance also influences their perception of how useful the technology is. The TAM, TAM-2, and TAM-3 are not specific to TEL or medical education. However, it is important to consider the impact of factors identified in these models when designing and implementing mandatory eLearning into postgraduate medical education.

Continuing education in therapeutics and safe prescribing is an important part of postgraduate medical education. Prescribing is one of the most frequent activities undertaken junior doctors in UK hospitals, yet trainees often report that they feel underprepared to prescribe following completion of their undergraduate education [13–17]. A study commissioned by the General Medical Council (GMC) found that trainee doctors prescribe with an average error rate of 10%, and concluded that training in practical prescribing should form part of undergraduate education [18]. In response to this recommendation, Health Education England (West Midland’s team) commissioned the development of SCRIPT—a web-based eLearning programme to promote safe, effective and rational prescribing (www.safeprescriber.com). The main aim of SCRIPT was to standardise prescribing education and encourage self-directed learning relating to prescribing and therapeutics. SCRIPT is now integrated into the training of Foundation trainee doctors in the West Midlands, and many other areas of the UK.

We used Kirkpatrick’s model of training evaluation [19] to examine Foundation trainees’ attitudes towards SCRIPT and its perceived impact upon prescribing behaviour in clinical practice.

## Method

### Background to the eLearning programme

SCRIPT eLearning comprises 48 modules across a range of specialities relating to prescribing and therapeutics. Modules include topics such as prescribing in Diabetic Emergencies, Renal Dysfunction, and the management of Sepsis. The modules were developed by a multidisciplinary team of healthcare professionals and are updated on a regular basis. Each module comprises a set of multiple choice questions, the main body of learning content, post-test questions, and suggested further reading. Following completion of the post-test questions, a certificate of completion can be viewed and downloaded by the user for their mandatory online learning portfolio (ePortfolio), which trainees are required to complete during the Foundation training programme.

Junior doctor rotations start in august of each year. In August 2011, it became compulsory for Foundation Year 1 (F1) doctors in the West Midlands to complete 16 specified modules within their first year of training. No other instruction about the order of module completion or time frame for completing modules was specified at that time. Foundation Year 2 (F2) doctors were also required to complete 15 modules of their choice. One of the aims of SCRIPT was to encourage self-directed learning relating to prescribing. As such, the programme does not emphasise assessment, however, there is an option for a pass mark to be assigned should a region wish to monitor the progress of trainees in this way. Bi-annual progression review meetings are held between Foundation trainees and their clinical tutor in early March and early June, for which the trainee must have prepared specific items in their ePortfolio for submission and evaluation as part of the annual review of competence progression within the Foundation training programme.

### Data collection

We conducted three semi-structured focus groups and four semi-structured interviews with 18 F1 and 17 F2 trainees. Focus groups lasted between 45 and 60 minutes, and interviews lasted between 10 and 15 minutes (30 minutes for the group interview). The structure of the focus groups and interviews were framed by two thematic components from Kirkpatrick’s model for evaluating the effectiveness of training [19]: 1) reaction to the training; and 2) behaviour change as a result of the training. A third theme of ‘Engagement’ was included based on previous research conducted to evaluate trainees’ completion of the learning over the course of their training year (Brooks et al. 2016). We developed a topic guide to structure each focus group and interview. A summary of themes and example questions are provided in Table 1.

**Table 1:** Example questions from the interview topic guide.

**Theme 1: Reaction**	What are your thoughts about prescribing education in the Foundation training programme?
	What are your thoughts about eLearning compared to other instructional methods (e.g. face-to-face teaching)?
**Theme 2: Behaviour**	Can you think of any examples of when information you have learned in SCRIPT has influenced your clinical practice?
**Theme 3: Engagement**	What factors influence your use of SCRIPT?

Two members of the research team conducted the focus groups. HB facilitated the session, and an independent qualitative researcher co-facilitated. HB conducted all of the interviews. Additional probes were used to explore participants’ responses in greater depth where appropriate, and questions were adapted as necessary for interviews or focus group settings. All sessions were recorded.

### Participants

To ensure the data collected were both credible and trustworthy [20], data were collected from both F1 and F2 trainee doctors at three different hospitals across the West Midlands region. The module completion requirements were the same for all the trainees involved in this research. Focus groups 1 and 2 were comprised of F1 and F2 trainees, respectively. Focus group 3 was mixed, comprising both F1 and F2 trainees. All participants provided written informed consent prior to the focus group or interview commencing.

### Ethical approval

The study protocol was approved by the University of Birmingham Ethical Review Committee [ERN_14-0746SB]. Research and Development approval was obtained from each of the hospital study sites.

### Analysis

Session recordings were transcribed verbatim and uploaded into NVivo 10 software to aid the organisation and analysis of data. Specifically, NVivo was used to store data transcripts, and as a means by which codes could be highlighted and collated, and from which the quotes in this manuscript are derived. The data were initially analysed and coded deductively [21], using the three themes described in Table 1 and then further explored for any new emerging themes. The coding process for each transcript involved carefully reading through each transcript and identifying phrases or sentences related to the three aforementioned themes (see Table 1). Also coded was any additional content deemed to be relevant to the research aims (i.e. related to attitudes towards SCRIPT or its impact on prescribing behaviours).

To enhance the credibility and trustworthiness of the data, analysis was undertaken by multiple researchers [20]. Two qualitative researchers undertook initial thematic analysis, one of whom was entirely independent from the SCRIPT eLearning project team. Following independent coding of a proportion of the transcripts, the two researchers then developed an initial coding schema. One researcher independently coded the remaining transcripts using this coding schema, and the second researcher reviewed the codes. Any disagreements were resolved through discussion. Following this, a group of expert clinicians (physicians and pharmacists) reviewed the codes and all of the transcripts; at least two clinicians independently reviewed each transcript. Minor amendments were made to the codes as necessary, after which the group agreed the major themes from the data. Once the group had achieved consensus about all codes and themes, a final arbitrator (a Professor of Pharmacy; JM) reviewed them. At this stage no changes were made to any codes or themes. At all stages of analysis, we engaged in negative case analysis, whereby we actively searched for comments that contradicted our coding schema, as recommended by Patton [20].

## Results

Data from 38 participants (site 1: n = 25; site 2: n = 5; and site 3: n = 8) were included in the analysis. Of the participants, 21 were in their first year of the Foundation programme (F1), and the remaining 17 in their second year (F2). Further details about the participants in the focus groups and interviews can be found in S1 Appendix.

Participants had studied at 15 different medical schools; one of which was outside the UK. Eighteen of the 38 participants studied at the University of Birmingham, and 21 within the West Midlands region. Further details about participants’ medical school education can be found in S2 Appendix.

**Fig 1:**
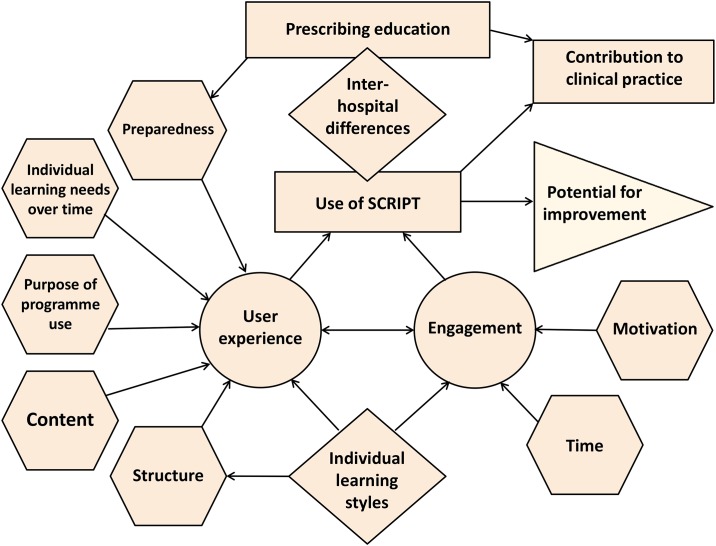
Schematic of the relationships between major themes and sub-themes.

The results revealed three major themes from the evaluation relating to (1) prescribing education in general, (2) factors affecting the user experience, and (3) factors affecting engagement with the programme. The major themes, sub-themes and codes can be found in S3 Appendix, and a schematic representation of the relationship between different themes and sub-themes highlighted though the data analysis is displayed in Fig 1. The main findings are discussed below.

### Prescribing education

Participants discussed the structure, strengths and weaknesses of undergraduate and post-graduate formal and informal prescribing education and the (potential) role of SCRIPT in both. Overall prescribing education appeared to impact upon preparedness to prescribe and directly contributed to participants’ clinical practice.

#### Undergraduate prescribing education

Many participants felt that their medical training adequately prepared them for practice from a theoretical perspective; however, they did not necessarily feel confident in their ability to prescribe in practice. Most participants agreed that the best way to learn was by working, an example of which is provided below:

“I felt as an undergrad, the general concepts were all covered. I think it was just, literally putting concepts into practice and actually knowing the doses and the times. And actually just writing the prescriptions and the speed at which I was doing it. That’s what I found was lacking at the start. But I don’t know if there is any way of doing that other than by working, really”

(F1, Focus Group 1).

SCRIPT is used primarily at the postgraduate level, however, greater preparedness to prescribe was an important factor that trainees identified positively contributed to their clinical practice. Some trainees felt that it would be useful to have online prescribing education such as SCRIPT available at the undergraduate level to supplement other prescribing education and prepare them for postgraduate medicine, as highlighted by an F1 from site 2.

“I think it would have been really useful at the undergraduate level, because we had quite good prescribing teaching in the final years, and practical sessions, but it would have been good in terms of, I think, in preparing starting FY1”

(F1, Focus Group 1)

Other trainees believed that SCRIPT would have been most valuable to them during the summer break between graduating from medical school and commencing clinical practice to help them feel more prepared in their prescribing knowledge and skills. In contrast, some participants were concerned about the possibility of having to complete mandatory educational activities during this ‘rest’ period prior to starting paid employment.

#### Postgraduate prescribing education

Participants provided descriptive information about their undergraduate and postgraduate prescribing education. Largely at the postgraduate level, participants differentiated between formal and informal prescribing education that they received. Types of formal education included lecture-based sessions, small group teaching, other mandatory TEL modules (for prescribing and other topics), and use of the British National Formulary (BNF). Informal education was provided in the form of on-the-job teaching from consultants, questioning other (often senior or more experienced) members of clinical staff such as pharmacists and nurses, and learning from doing the work themselves. S4 Appendix contains the postgraduate learning resources, both formal and informal, that participants identified.

#### Inter-hospital differences

Differences emerged in how SCRIPT was merged into existing training, including the potential rewards for completing SCRIPT modules. At site 1, trainees were expected to complete specified SCRIPT modules in time outside of paid employment before attending a three-hour teaching session expanding on the material from that module. In contrast, at site 2, some formal teaching was provided but this did not relate directly to SCRIPT, and if trainees were unable to attend compulsory teaching sessions they could receive credit by completing SCRIPT modules and providing evidence via certificates of completion. At site 2, no specific time was provided in which to complete SCRIPT modules and no credit could be obtained for their completion. Trainees identified both benefits (e.g. reinforcement at site 1) and drawbacks (e.g. repetition at site 1) of the different strategies.

### User Engagement

Engagement with SCRIPT was dependent on trainees’ motivation to complete modules and the time available to complete modules. As shown in Fig 1, these factors contributed learners’ use of SCRIPT and their experience with the programme.

#### Motivation

The mandatory nature of SCRIPT appeared to impact upon many trainees’ perceptions of the programme. They acknowledged that much of the content was relevant, useful, and that the programme was well-structured and easy to use. However, SCRIPT was viewed by many as a tick-box exercise, with mandatory modules primarily being completed to ensure that both interim and annual progress reviews were passed.

“So I think the primary factor obviously has to be the fact that it’s compulsory. I think that’s obviously the thing that drives you the most to do it”

(F2, site 1)

Some participants perceived that their supervisors and clinical educators did not value SCRIPT and did not often discuss trainees’ progress at review meetings, unless the trainee had failed to complete the required mandatory components. As one F1 trainee (site 1) highlighted, “Our tutors just don’t care. They have more important things to do”. Likewise, learners did not always value SCRIPT. For example, one F2 trainee at site 3 felt that as there was no pass mark for the modules this devalued the learning tool:

“If we’re genuinely learning, and if we’re genuinely training, then there should be some level that we’re all deemed to be acceptable. Otherwise, what’s the point in carrying a certificate if I just got 10% and I clicked through the whole thing. It makes no sense. For this to be validated and for me to actually feel that I’ve come out of this with a genuine understanding of what I’ve learnt, it would be nice to have a formal assessment”

(F2, site 3)

Nonetheless, despite the mandatory nature of SCRIPT and this sometimes being the primary motivation for completing the learning, many users still valued the programme as useful learning tool. For example, an F2 from site 1 stated, “to be honest, the things that are mandatory should always be useful, and of the things that are mandatory SCRIPT is the most useful thing that we do”.

#### Time

TEL should afford the opportunity for flexibility in the time and place of learning, however, time was an important and limiting factor for trainees when completing SCRIPT modules. Participants commented on the mandatory nature of SCRIPT but admitted to completing the majority of modules at home during free time. The majority of trainees were negative about this as motivation to complete modules after a full day at work was low. For example, an F1 trainee (site 2) stated, “you don’t want to go home, after doing three nights in a row and be doing SCRIPT, it’s just not what you’re going to do”. On some occasions trainees found it possible to complete modules during paid employment; however completing modules during this time was not always considered ideal as if trainees’ completed modules while sitting at a computer on the wards they were often interrupted, or when completing modules in free time during a night shift they were often tired and unable to focus.

In general, participants agreed that the lack of protected time to complete modules during paid employment resulted in disengagement from the programme and a tendency to rush over module content. An F2 (site 3) highlighted, “…as F2s this past week we’ve just panicked. And we’ve done all 25 SCRIPT modules as F2s in three days of literally sitting there just clicking”. This data was supported by another F2 (site 3), who said, “we sat as a house–I live with seven other F2s –and over the weekend we just sat there and we just clicked. We just clicked. And we didn’t care, in all honesty”. These findings provide explanation for the findings from previous evaluative work conducted by Brooks et al [8], in which the authors found that some trainees were completing modules very quickly and close to assessment submission deadlines.

In contrast, one F2 trainee acknowledged that continued professional development (CPD), which is likely to involve unpaid work outside of paid employment, is a necessity for a successful career in medicine. As such, he conceded that is may be necessary to view SCRIPT as a part of this CPD and to accept that it may be completed in one’s own time. He stated:

“if you can do it in work, great but I think all of us know and accept that we have to do things outside of our, you know, normal working hours to progress in the career anyway. So it’s how you organise your own free time”

(F2, site 1)

### User Experience

The user experience affected engagement and use of SCRIPT, and as such, it indirectly contributed to clinical practice. A number of factors appeared to influence the user experience, including the rationale for using the programme, the relevance of content, structure and purpose of using the programme, as well as the individualisation of the learning to different learning styles and learning needs over time. Preparedness also appeared to impact upon the user experience.

#### Reason for use

Trainees stated a number of rationales for using SCRIPT, including: to provide core prescribing knowledge or revision on previously learned topics; to obtain feedback on their own prescribing knowledge; to fill gaps in knowledge that they were unable to gain from other resources; as a tool to signpost to other useful resources; and to fulfil the mandatory training requirements. SCRIPT was used as a reflective (e.g. when a challenging prescribing situation was encountered trainees would refer back to SCRIPT to help clarify their knowledge about the situation) and proactive (e.g. prior to starting a new rotation trainees would prepare by completing modules directly related to that rotation in preparation) learning tool. This suggests that despite its mandatory nature, SCRIPT provided additional learning opportunities for trainees. For example, one F1 (site 1) suggested, “having done SCRIPT modules I’ve thought about situations where, ‘oh I would have done that differently’”. Some trainees completed modules in a proactive nature to help them prepare for upcoming rotations. One trainee stated:

“So for example, before I started this rotation–this is a diabetes ward–before I started this job, I did the diabetes SCRIPT module for that reason, not because it’s compulsory, just because I thought it would be useful”

(F2, site 1)

Overall, there appeared to be a desire to use the programme beyond the mandatory requirements, but due to a lack of time this was not always possible.

#### Content

Despite issues with engagement due to the mandatory nature of modules and a lack of protected time in which to complete them, when users did engage with the learning content they found this to be a generally positive experience. This was a consistent message regardless of the site, prescribing system used (paper s. electronic) or level of trainee (F1 or F2). Trainees perceived the content to be relevant and useful, and provided trainees with reassurance that they had acquired an appropriate level of knowledge. Some SCRIPT content was duplicated with content from undergraduate or post-graduate curriculum, which some trainees viewed as unnecessary repetition and others viewed as useful revision. Despite being a primary research question, trainees perceived the direct impact of SCRIPT on prescribing behaviours in clinical practice to be minimal. However, many participants provided examples in which knowledge gained from SCRIPT had been useful in clinical practice, from using knowledge in their own prescribing to having the confidence to question other doctors’ prescriptions. Below is an example from an F1 trainee:

“You wouldn’t like, I can’t think of specific examples. But let’s say someone’s got acute kidney injury and you review your chart and you’re like, are looking for nephrotoxic medication. I mean, having done some of the SCRIPT modules, I’m just more…it’s probably helped me pick up nephrotoxic medication”

(F1, site 1)

In particular, a number of participants suggested that clinically relevant content, as opposed to theoretical content, was most useful, as it was possible to transfer the knowledge directly to clinical practice. This suggests that SCRIPT appears to enhance prescribing knowledge, which in turn contributes to enhance prescribing in clinical practice in some cases.

#### Structure

Differences existed in trainees’ perceptions about the effectiveness of eLearning as an educational tool. Trainees highlighted a lack of consideration for individual learning styles and some learners’ preference for other modes of teaching to support their own learning. Though many trainees enjoyed the ability to work at their own pace and completed the modules in a favourable environment at a time that suited them, not all trainees liked eLearning. For example, one trainee (F1, site 2) highlighted, “I think ideally, I mean, I like to talk to people so I’d rather be taught”.

Some trainees believed that the modules were too long, which had a negative impact on the user experience and subsequently engagement. For example, as one F1 (site 1) highlighted, “The thing is with SCRIPT is that it’s quite long. So I tend to either have to break it up or just skip through the last half”. An F2 trainee from the same site added, “They keep telling you in all these theories of teaching that more than half an hour and nobody listens, and again and again everything’s longer than half an hour”. However, other trainees disagreed, stating that it is perfectly easy to exit the programme and complete modules later, as the programme automatically saves progress through modules. For example, an F2 (site 1) argued “… it saves your progress anyway, so if it takes you a while you can just do half one day and continue the next day”.

#### Learning needs

Trainees’ also highlighted the need for the learning resource to complement their own learning needs at a given point in time, depending on the patient population they are working with, their prior knowledge and prescribing experience (e.g. there will be different learning needs in the F1 compared to the F2 year). For example, an F2 trainee stated:

“Depending on what job you’re in, you’ll be prescribing different things on a more regular basis. So it varies depending, you know, every time you change jobs there’s more to learn”

(F2, site 1)

The availability of a wide variety of (compulsory and optional) modules was considered useful to complement learning needs of individual trainees, however trainees sometimes found it frustrating that mandatory modules incorporated information that they already knew.

“Yeah, I did a module recently and it showed you how to do really basic things that I’ve been able to do for about 7 years. Just like, how to give an IM [intra muscular] injection or a Sub-cut[aneous] injection, and I was just sat there like, ‘you don’t really need to recap that. I’ve got that down, thanks’”

(F2, site 3)

This feedback suggests the need to consider a more flexible approach to learning, potentially selecting a smaller group of core modules but allowing the learner to choose additional modules that are relevant to them at a given time in their Foundation training years.

### Potential for Improvements

Throughout the focus groups and interviews, participants provided suggestions for ways to improve the programme and its integration into training. The most common suggestions were to:

- Provide access to the programme from the undergraduate level through the Foundation training years and beyond
- Provide dedicated time to complete modules during paid employment
- Allow trainees greater choice in module selection, to avoid duplication of learning or having to learn information that may be irrelevant to that particular trainee at that particular time
- Decrease the length of certain modules to provide bite-size chunks of information that allow trainees to complete the learning in a reasonable time (e.g. 30 minutes)

Implementing these suggested improvements may help to improve trainees’ perceptions of SCRIPT and their motivation to engage with the programme for reasons other than completing mandatory modules.

## Discussion

In this study, we evaluated UK Foundation trainee doctors’ attitudes towards mandatory TEL modules to improve prescribing competency. We used the “reaction” and “behaviour” levels of Kirkpatrick’s model of training evaluation as a framework to guide this qualitative evaluation, and expanded upon previous findings from Brooks et al [8]. Concerning trainees’ attitudes towards SCRIPT eLearning, in general trainees perceived it to be useful, relevant and easy to use, which supports previous research indicating the positive impact of eLearning in medical education [7]. Trainees used SCRIPT to fulfil the mandatory requirements of the Foundation training programme. However, the programme was also used to fill gaps in knowledge, gain feedback, as a revision tool and to signpost to other learning resources. Barriers to engagement and use of the programme included time and a lack of reward or value being placed upon the learning. These data highlight the need to provide more individualised content and structure, which may not be possible through eLearning [9]. It appears important to provide protected teaching time in which trainees can complete the mandatory components. Furthermore, it is imperative to consider carefully the length of modules during the development of future modules, in order to optimise engagement. Trainees suggested a need for greater choice and flexibility in when and which modules are completed, including the potential for expansion to the undergraduate curriculum (which is something that has already been implemented in some medical schools).

The data suggest that there may be a small direct and larger indirect impact of SCRIPT on prescribing behaviours in clinical practice. The programme appears to complement the various other formal and informal sources of prescribing education available to doctors in early training. Hospitals used different strategies to integrate SCRIPT, which may have affected trainees’ perceptions of SCRIPT (e.g. due to the duplication of learning from SCRIPT modules to teaching sessions) and the impact of SCRIPT on prescribing behaviours. Therefore, although there may be differences in learning preferences [9], SCRIPT has a clear role as a mandatory component of postgraduate medical education, and can be used to complement other learning resources, all of which may have an impact (direct or indirect) on prescribing in clinical practice.

### Implications for educators

The findings from this study highlight the importance of developing effective prescribing education that engages learners whilst providing relevant information within a minimal timeframe. First, postgraduate medical educators should afford Foundation trainees the opportunity to complete as much as possible of any mandatory TEL during paid employment and within protected study time allocated throughout the Foundation programme. Given that this opportunity may currently differ across hospitals it is also important to standardise requirements of TEL and opportunities to complete TEL across hospitals. Second, educators should consider the value that the learning holds for both learners and employers or supervisors, and the impact that this may have on their motivation, particularly when the learning is mandatory. By placing value on the learning (e.g. pass mark; supervisor checks on progress) this may help motivate learners to engage with the content more effectively. Similarly, it may be possible to enhance engagement by providing a positive user experience, for example: by creating innovative and interesting content that will help to provide both core knowledge and additional information to suit individual learning needs; allowing trainees to have some choice in the mandatory learning that they complete; and ensuring that modules are of an optimal length. It may also be useful to provide an opportunity for all undergraduate learners to access learning resources prior to commencing postgraduate training to facilitate greater preparedness at the start of clinical practice. It is also important to consider the potential negative consequences of placing additional demands on the undergraduate curriculum, and to provide education that is proximate to practice. As such, it may not be feasible to mandate learning at the undergraduate level but simply provide an opportunity for undergraduates to access such additional resources. Finally, the findings from this evaluation highlight the importance of evaluating learning resources such as SCRIPT, so that improvements can be made in order for learning resources to be more effective. Many of the findings from this study, particularly those related to engagement and motivation, may be taken forward not only by postgraduate medical educators in the UK, but by educators from other academic or professional levels or disciplines, including those in countries outside the UK.

### Limitations and future research

As previously described, although the requirements for completing mandatory SCRIPT modules was consistent across the three sites evaluated, there were variations in how these were integrated into the training programme. At site 1, trainees have face-to-face teaching during protected time related to and extending upon specific SCRIPT modules, which trainees are expected to have completed in advance. At sites 2 and 3, modules are completed at times chosen by the trainee (within required institutional deadlines) and there is no directly related taught prescribing education. These differences may have had an impact on trainees’ prescribing knowledge and also their perceptions about both postgraduate prescribing education (they may not have enjoyed the taught classes as they had already learned the content through the SCRIPT modules prior to the class) and/or SCRIPT modules (they may not have managed to complete the SCRIPT module prior to the class and so felt that their learning was duplicated through the eLearning programme). Duplication of learning may have been due to the structure of their formal prescribing education rather than a flaw of SCRIPT. In this study we did not examine in depth the distinct effects of the various methods used to integrate SCRIPT into formal education on trainees’ perceptions of SCRIPT or its impact on their prescribing in clinical practice. Future research should address these issues.

## Conclusion

SCRIPT eLearning is a mandated online prescribing learning resource for Foundation trainee doctors. Here, we have found that trainees perceived the tool to be a useful learning resource but that a lack of dedicated time to complete the learning was a barrier to engagement. In general, SCRIPT was perceived to have an indirect, positive impact upon prescribing behaviours in clinical practice. Educators should consider various factors in the design and implementation of mandated eLearning for postgraduate medical education, including individual learning styles and needs, and the impact of time and motivation on user engagement and satisfaction.

## Supporting Information

S1 Appendix Overview of participant characteristics by focus group/interview. (DOCX)

S2 Appendix Institutions where participants’ studied their undergraduate medical degree. (DOCX)

S3 Appendix Themes, subthemes and codes. (DOCX)

S4 Appendix Formal and informal prescribing education resources. (DOCX)
