# Supplementary material for: Perceptions and Impact of Mandatory eLearning for Foundation Trainee Doctors: A Qualitative Evaluation
Source: PLoS One. 2016 Dec 22;11(12):e0168558. doi: 10.1371/journal.pone.0168558 (PMC5179017; doi:10.1371/journal.pone.0168558)
Supplement: S1 Appendix — (DOCX) [file pone.0168558.s001.docx]

**S1 Appendix: Overview of participant characteristics by focus group/interview**

| **Focus Group/ Interview** | **Site** | **Number of participants** | **Number of F1 doctors** |
| --- | --- | --- | --- |
| Focus group 1 | 1 | 15 | 15 |
| Focus group 2 | 1 | 10 | 0 |
| Interview 1 | 2 | 2 | 1 |
| Interview 2 | 2 | 1 | 1 |
| Interview 3 | 2 | 1 | 0 |
| Interview 4 | 2 | 1 | 1 |
| Focus group 3 | 3 | 8 | 3 |
| ***Total*** | | **38** | **21** |
