# Supplementary material for: Perceptions and Impact of Mandatory eLearning for Foundation Trainee Doctors: A Qualitative Evaluation
Source: PLoS One. 2016 Dec 22;11(12):e0168558. doi: 10.1371/journal.pone.0168558 (PMC5179017; doi:10.1371/journal.pone.0168558)
Supplement: S2 Appendix — (DOCX) [file pone.0168558.s002.docx]

**S2 Appendix: Institutions where participants’ studied their undergraduate medical degree**

| **Undergraduate Institution** | **Number of participants** |
| --- | --- |
| Bristol University | 2 |
| Imperial College London | 3 |
| International | 1 |
| Keele University | 1 |
| Liverpool University | 1 |
| Peninsula | 2 |
| Southampton University | 2 |
| University College London | 2 |
| University of Birmingham | 18 |
| University of Leicester | 1 |
| University of Manchester | 1 |
| University of Nottingham | 1 |
| University of Oxford | 1 |
| University of Sheffield | 1 |
| University of Warwick | 2 |
