# Supplementary material for: Perceptions and Impact of Mandatory eLearning for Foundation Trainee Doctors: A Qualitative Evaluation
Source: PLoS One. 2016 Dec 22;11(12):e0168558. doi: 10.1371/journal.pone.0168558 (PMC5179017; doi:10.1371/journal.pone.0168558)
Supplement: S3 Appendix — (DOCX) [file pone.0168558.s003.docx]

**S3 Appendix: Themes, subthemes and codes**

| **Theme** | **Sub Theme** | **Code** |
| --- | --- | --- |
| **Engagement** | Motivation | Perceived value to employer/supervisor  Value to user  Reward  Mandatory nature  Other motivators |
|  | Time | Personal time  Time during paid employment  Engagement with content |
| **User Experience** | Reason for use | Feedback from system  Core prescribing knowledge  Gap filling  Mandatory requirements  Proactive learning  Reflective learning  Revision  Signposting  Supplementary learning |
|  | Content | Duplication of learning  Relevance of content  Contribution to clinical practice  Specific module relevance  Paper Vs. electronic prescribing  Reassurance  Usefulness |
|  | Structure | Interactivity  Usability  Module length  Individual learning styles  eLearning as a platform |
|  | Learning needs | F1 Vs F2  Individual learning needs  Targeted learning  Relevance at different time points |
|  | Potential for Improvement | Access  Bite size  Module choice  Protected time  Optimal module length  Other improvements |
| **Prescribing education** | Undergraduate Prescribing Education | Formal UG prescribing education  Informal UG prescribing education  Preparedness |
|  | Postgraduate Prescribing Education | Formal PG prescribing education  Informal PG prescribing education  Experience  Continued Professional Development  Other learning resources  Monitoring |
|  | Inter-hospital differences | Inter-hospital differences |
