# Supplementary material for: Perceptions and Impact of Mandatory eLearning for Foundation Trainee Doctors: A Qualitative Evaluation
Source: PLoS One. 2016 Dec 22;11(12):e0168558. doi: 10.1371/journal.pone.0168558 (PMC5179017; doi:10.1371/journal.pone.0168558)
Supplement: S4 Appendix — (DOCX) [file pone.0168558.s004.docx]

**S3 Appendix: Formal and informal prescribing education resources**

| **Formal learning resources** | **Informal learning resources** |
| --- | --- |
| - Hospital guidelines/Trust protocols | - Consultants |
| - National Institute for Health and Care Excellence (NICE) Guidelines | - Other senior doctors (e.g. Registrar) - Pharmacists - Senior nursing staff - Other junior doctors - Experience from own prescribing |
| - Hospital intranet page |  |
| - Link from electronic prescribing system |  |
| - eLearning for Health |  |
| - British National Formulary (BNF; paper version or online App) |  |
| - British Medical Journal (BMJ) Learning |  |
| - SCRIPT eLearning |  |
| - Specific resources independently created by specific ward (e.g. “Palliative Book”) |  |
| - Pharmacology text book |  |
